# Preliminary Investigation of a Potential Optical Biosensor Using the Diamond™ Nucleic Acid Dye Applied to DNA and Friction Ridge Analysis from Fingerprint Traces

**DOI:** 10.3390/bios14110546

**Published:** 2024-11-11

**Authors:** Martyna Czarnomska, Aneta Lewkowicz, Emilia Gruszczyńska, Katarzyna Walczewska-Szewc, Zygmunt Gryczyński, Piotr Bojarski, Sławomir Steinborn

**Affiliations:** 1 Faculty of Mathematics, Physics and Informatics, University of Gdansk, ul. Wita Stwosza 57, 80-308 Gdańsk, Poland; aneta.lewkowicz@ug.edu.pl (A.L.); emilia.gruszczynska@phdstud.ug.edu.pl (E.G.); 2 Institute of Physics, Faculty of Physics, Astronomy and Informatics, Nicolaus Copernicus University, ul. Grudziądzka 5, 87-100 Toruń, Poland; kszewc@umk.pl; 3 Department of Physics and Astronomy, Texas Christian University, 2800 S. University Dr., Fort Worth, TX 76129, USA; z.gryczynski@tcu.edu; 4 Faculty of Law and Administration, University of Gdansk, ul. Jana Bażyńskiego 6, 80-309 Gdańsk, Poland; slawomir.steinborn@prawo.ug.edu.pl

**Keywords:** Diamond™ Nucleic Acid Dye, touch DNA, fingerprint, evidence, forensic science, identification

## Abstract

Developments in science and technology lead to an increasing use of scientific evidence in litigation. Interdisciplinary research can improve current procedures and introduce new ones for the disclosure and examination of evidence. The dactyloscopic trace is used for personal identification by matching minutiae (the minimum required may vary by country) or for extracting DNA material from the trace under investigation. The research presented in this article aims to propose the merging of two currently used personal identification methods, DNA analysis and dactyloscopic trace analysis, which are currently treated as separate forensic traces found at a crime scene. Namely, the forensic trace to be analyzed is the dactyloscopic trace containing DNA, and both sources of information needed for identification are examined as one. Promega’s Diamond™ Nucleic Acid Dye, presented as a safe alternative to ethidium bromide, works by binding to single- and double-stranded DNA and is used to visualize the separation of material in a gel and to detect DNA in forensic samples. Spectroscopic studies as absorption and emission spectra and fluorescence microscopy observations presented in our research confirm that Diamond™ Nucleic Acid Dye can also be used to visualize fingerprints on non-absorbent surfaces and that combining the two methods into one can significantly increase the evidential value and contribute to the design of an innovative fast-acting optical biosensor.

## 1. Introduction

The development and implementation of new methods in forensic science is driven by scientific progress. Improving the criminal justice system requires that judges, law enforcement agencies, and forensic scientists work together to achieve accurate results from challenging samples [1]. Technological and methodological advances in the examination of evidence mean that judges should be more familiar with new examination methods and keep abreast of scientific advances [2]. The application of science in the criminal justice process is not ‘mere facts or asserted truths’ but rather to be part of a narrative, science must be presented as propositions or objects that help us to determine which narrative is more plausible. Scientific evidence must obey admissibility frameworks and rules designed to meet the needs of the law [1]. 

Fingerprinting is part of the scientific application of criminal justice, the most common use of which is to link a person to a criminal record [3]. The procedure is known as ‘friction ridge analysis’ and is based on comparing the ridge structures of fingerprints [4]. Current identification processes are based on either DNA analysis or fingerprint analysis through the analysis of minutiae systems. The uniqueness of the individual and immutability of the fingerprint, or more precisely the ridge pattern, has always been considered the best reference for personal identification in forensic science [5]; moreover, fingerprints differ even in twins sharing the same DNA [6]. In the United States, for example, people joining the military or applying for government jobs are required to submit their fingerprints for comparison with criminal records held by the FBI—the Federal Bureau of Investigation [3].

Fingerprints found at a crime scene are divided into three groups. The first is indent fingerprints, which are impressions made by the fingertips, for example, in candle wax. The second is visible fingerprints, which are, for example, stained with a substance such as blood, and the third and the most common type of fingerprint evidence are latent fingerprints, which are the unintentional impressions left by the fingers on the surfaces of objects [7]. Latent fingerprint matching remains a challenging problem due to the poor quality of ridge impressions and the small area of the finger [8], but still, they are the most frequently sampled evidence in forensic investigations [9,10]. The examination of latent fingerprints involves several main points, such as recognition, identification, individualization, and verification [11], and the literature gives us a recommendation that fingerprinting should always be undertaken before DNA analysis [12]. Forensic science relies on many different types of biological evidence that can provide a person’s genetic profile, including blood, saliva, hair, bones, urine, and the object of this paper—fingerprints [13,14,15,16,17,18,19].

A 1997 study found that it is possible to obtain a genetic profile from objects held or touched by offenders and has been used to prove attempted murder, rape, and drug trafficking [20]. Further research in 2019 has shown the possibility of extracting DNA from fingerprints taken from different surfaces using tape and gel lifters [21], but there is a significant loss of material when collecting DNA evidence, with gel lifting generally being the least destructive [22].

Biological material collected from crime scenes is a valuable source of genetic information, but the low quality and limited amount of DNA extracted from these samples can present a significant challenge for further analysis. Degradation, decomposition, and contamination can significantly affect DNA analysis [23]. Due to the many difficulties associated with extracting material in the form of DNA from fingerprints, the impact of environmental factors on such material and, most importantly, determining the exact source of DNA found on prints—the issues surrounding the use of fingerprint DNA—are still under investigation. The use of such traces for genetic identification is a crucial component of forensic science that requires further development, highlighting the advantages, disadvantages, and limitations of DNA analysis from fingerprints, as well as finding new methods for application in forensic science. Scientific studies have confirmed the great potential of obtaining a complete genetic profile from fingerprints and their potential use in a judicial context, with special handling of the material and a cautious, careful approach to the process [24]. Therefore, research in this manuscript presents the use of Diamond™ Nucleic Acid Dye (DD) to test a single piece of evidence in two ways to increase its evidential value. 

DD is proposed as a safe alternative to ethidium bromide, a common, well-known, and toxic fluorescent dye, with DD showing no toxic properties at the recommended dilution of 1:100,000 [25]. In addition, the use of DD has been evaluated in very broad types of studies to confirm the presence of DNA on a swab [26], to assess hair roots for the presence of DNA [27], to assess the viability of the sample for STR analysis [28], to evaluate for use in quantitative PCR applications [29], and to locate fingerprint and touch DNA on non-porous objects [30], showing great potential for this fluorescent dye. 

Methods that combine two types of analysis of fingerprints using Diamond Nuclei Acid Dye—DNA examination and dactyloscopic evaluation by friction ridge analysis—are not currently available in the literature due to the lack of available techniques. The literature delivers information on a very wide range of fluorescent dyes to visualize DNA in biological samples—Ethidium Bromide, SYBR Green I, DAPI, Cyanoacrylate, Rhodamine 6G, and RedSafe (RS)—but all of them demonstrate disadvantages in use, such as high toxicity, inability to penetrate the cell membrane, low efficiency, their interference with further examination of DNA, or their need for more DNA for detection than DD [31,32,33,34]. The approach we present shows the possibility of first visualizing the trace using the fluorescence phenomenon and then demonstrating the lack of influence of the dye on the further handling of the material.

Over the past few years, research into the use of Diamond™ Nucleic Acid Dye has had various application objectives, although they mainly concern biological aspects of the use of the dye. These have ranged from how to apply the dye on the specimens and modify the concentrations of the solution [35] to analyzing processes for detecting latent DNA and developing cell localization capabilities [36], the impact of dye application on subsequent immunoassays, DNA extraction, profiling, or the possibility of using PCR after dye application [37]. Other applications include monitoring the reduction of cell deposition during multiple contact [38], analyzing the most compatible surface for the dye [39], the ability of Diamond™ Nucleic Acid Dye to examine the persistence of cells on different surfaces after immersion in water [40], and the effect of tape lifting on the recovery of touch DNA [41]. 

Thus, we see a great diversity of research directions, but none of the above literature presents the results of the spectral characterization of dyes. Fluorescence microscopy is only one of the possible analyses of DNA material stained with Diamond™ Nucleic Acid Dye. A thorough spectral characterization of the compound absorption and emission spectra can give us valuable information about the dye analyzed, the complexes it forms with DNA, how it binds, and much more. The research presented here is the first step towards designing biosensors for the most common form of DNA left at a crime scene—touch DNA.

## 2. Materials and Methods

### 2.1. Materials

#### Preparation of the Solutions

The Diamond™ Nucleic Acid Dye solution was made according to the manufacturer’s instructions, i.e., diluted 1 × 100,000 in TBE buffer (Solution containing 89 mM Tris, 89 mM boric acid, 2 mM EDTA).

Deoxyribonucleic acid from herring sperm was dissolved in distilled water at a DNA concentration ~5 µg/µL—“reference DNA”. 

The 10× concentrated TBE buffer was diluted with distilled water to 1× concentration. Information on all reagents is presented in Table 1. 

### 2.2. Methods

#### 2.2.1. UV-VIS/Fluorescence Spectral Measurements

UV-VIS spectral studies were measured with Shimadzu double-beam UV-Vis spectrophotometer UV-1900i in the Spectrum Mode. Software—LabSolutions UV-Vis.

Shimadzu Europa GmbH, Duisburg, Germany.

Fluorescence spectral studies were measured with Horiba Jobin Yvon, model FluoroMax 4 TCSPC in the Emission Mode. Software—FluorEssence V3.

HORIBA Europe GmbH, Oberursel, Germany.

Results presented on the spectra in this study are averages of samples from all study participants.

#### 2.2.2. Fingerprint Collection/Observation

Fingerprints were taken from a participant—10 people, 2 series every 24 h for 2 days—who had abstained from food, drink, and hand hygiene (for at least 1 h before fingerprinting). The fingerprint was taken by placing the index finger on a sterile microscope slide. The fingerprint left on the slide was stained with Diamond™ Nucleic Acid Dye solution and then observed using an Olympus SZX16 stereo microscope with fluorescence—filter GFP—after drying.

**Figure 1:**
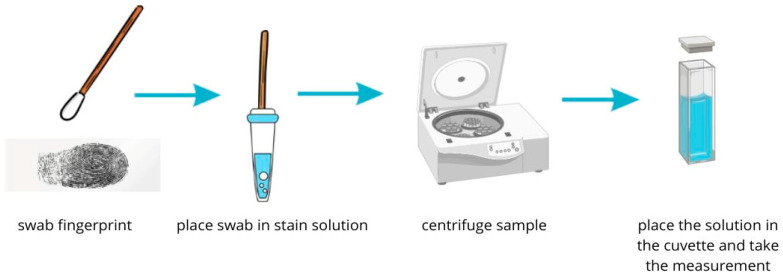
Fingerprint collection procedure.

The fingerprints were a source of the examined “fingerprint DNA” samples, according to the procedure in Figure 1.

#### 2.2.3. Direct PCR

Direct PCR reaction was performed on a Mini PCR^®^ mini8 thermal cycler (Minipcrbio Cambridge, MA, USA). The fingerprint was applied to a microscope slide, then the material was collected with a sterile swab, placed in a 2 mL Eppendorf, filled with distilled water, and centrifuged at 1000 rpm for 3 min. Subsequently, 50 µL of material was collected from the bottom of the Eppendorf, transferred to a 0.2 mL Eppendorf, and direct PCR reagents from the Direct Tissue PCR Kit were added. The Eppendorf was placed in a thermal cycler, and the reaction was performed, producing “fingerprint DNA after PCR” samples.

#### 2.2.4. Research Permission and Ethics Declarations

The study was approved by the Ethics Committee of the University of Gdańsk. Full informed and written consent from the participants was obtained before the initiation of the study for study participation and publication of the pictures used in the manuscript. The experimental protocol was approved by the University of Gdańsk and all methods were performed according to the relevant guidelines and regulations.

## 3. Results and Discussion

**Figure 2:**
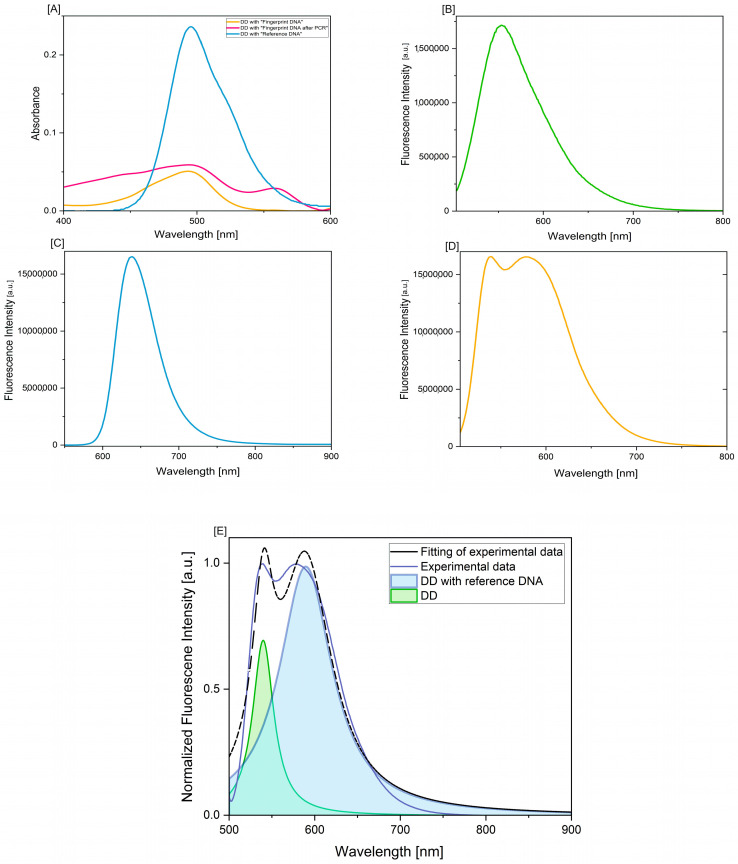
(**A**) Absorption spectrum of DD, DD with DNA from fingerprints, and DD with DNA from fingerprints after PCR; (**B**) emission spectrum of DD-excitation 494 nm; (**C**) emission spectrum of DD with reference DNA-excitation 505 nm; (**D**) emission spectrum of DD with DNA from fingerprint–excitation 494 nm; (**E**) deconvolution of the emission spectrum of Diamond™ Nucleic Acid Dye.

The absorption spectra—Figure 2A and Table 2—confirm the possibility of detecting fingerprint DNA; in addition, a signal enhancement was obtained after the amplification step. The integrated area under the absorption band demonstrates the possibility of obtaining evidence in the form of DNA from fingerprints with an intensity of the absorption band comparable to DNA references. Integration of the absorption spectral region in the 400–600 nm range is suitable for comparing the dye binding efficiency of the measured samples. The concept of spectral integration is that the area of a given absorption spectrum is proportional to the number of (equivalent) photons that constitute the spectra examined. In practice, this is determined by the integration curve. The integration curve appears as a series of steps, the height of each step being proportional to the area of the corresponding absorption maximum and thus to the number of photons responsible for the absorption. It should be borne in mind that it can be challenging to determine precisely where the integration measurement begins and ends, and the coefficients should not be assumed to be exact integers.

An absorption band maximum was obtained at 494 nm for DD with fingerprint DNA, and DNA with fingerprint DNA after PCR and at 505 nm for DD with reference DNA. The differences in the intensity of the absorption band maxima for DD with reference DNA and DD with fingerprint DNA are due to the presence of other substances in the case of fingerprint DNA and non-isolated DNA material as opposed to reference DNA. For the absorption band obtained for the PCR fingerprint DNA sample, a second maximum was obtained at 558 nm, which characterizes the spectroscopic properties of the fluorescent compound present in the Direct PCR kit.

The emission spectra of the Diamond™ Nucleic Acid Dye solution (2B) are consistent with the manufacturer’s data and the literature [42] and have their maximum at around 555 nm. When analyzing DD spectra with reference DNA (2C), we see a shift of the spectrum towards the long wavelength and the emission maximum is at 639 nm.

In the case of a DD spectrum with DNA from a fingerprint swab (2D), the spectrum has two maxima, the first at 540 nm and the second at 578 nm. The slight differences in the position of the fluorescence band maximum for the DD complex with reference DNA and the DD complex with fingerprint DNA are due to the complexity of the matrix, which is a sweat-fatty substance. Also, the DNA from fingerprints is not isolated and contains other fingerprint components that may interfere with the fluorescence of the DD-DNA complex. On fingerprints, we find substances produced by sweat and sebaceous glands located in the dermis layer of the skin: eccrine and apocrine secretions are a mixture of various inorganic–organic compounds such as NaCl, urea, and amino acids, while sebaceous secretions consist of compounds such as glycerides, fatty acids, wax esters, squalene, sterols, and sterol esters [30,43,44,45,46,47,48].

Figure 2E presents the deconvolution performed with actual spectral profiles of single-component reference compounds, and Gaussian fitting of the spectra indicates the presence of two forms-free dye and those that bound to DNA. These values confirm that the complexation reaction of DD with DNA occurred and that the DNA sample obtained from the fingerprints can be used for further studies.

Due to the small amount of DNA and complexity of the matrix, we have to choose a good enhancement method to prevent contamination and loss of DNA [12,34] as results show high variability in the amount of extracted material depending on the method and the surface from which the trace was collected [49]. Important aspects in the visualization of DNA using fluorescent compounds are high contrast, sensitivity, selectivity, but also low toxicity and consideration of factors such as the effect that harmful UV light can have on DNA yield, but also on our eyes and skin as users of visualization procedures [50,51,52].

**Figure 3:**
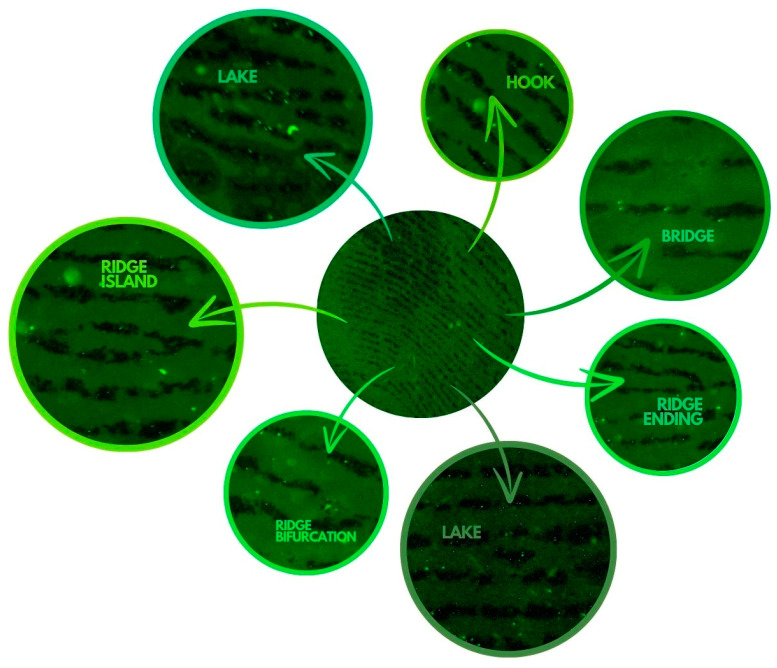
Visualization of characteristic latent minutiae with Diamond™ Nucleic Acid Dye.

**Figure 4:**
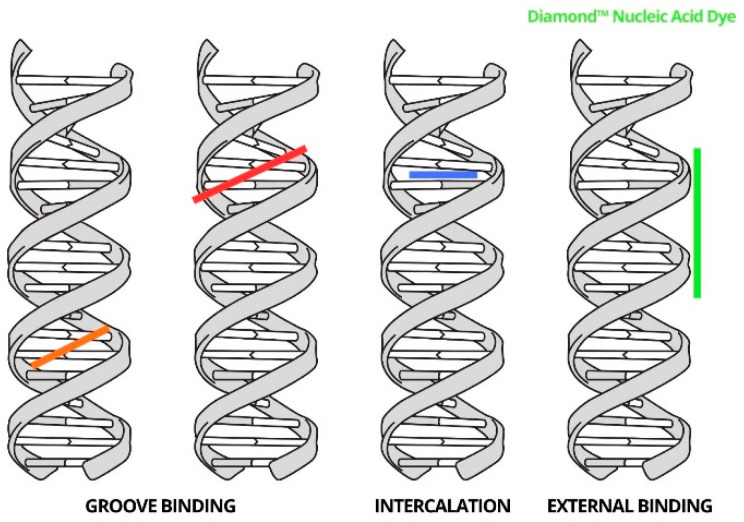
Fluorescent dye binding to DNA model [53,54].

The fluorescence microscopy application in our study confirmed that the DD dye can be used simultaneously to visualize fingerprints—Figure 3—and does not interfere with the fluorescence of fingerprints, while allowing DNA analysis. The fluorescence of fingerprints on a non-porous surface with the DD solution enabled the identification of characteristic arrangements of minutiae in the fingerprint. Figure 4 shows examples of lake, hook, bridge, ridge island, ridge bifurcation, and ridge end minutiae in a single print stained with DD dye solution that could be used for individual identification. An important conclusion is that the DD dye can be used to label DNA in fingerprints and prepare samples for further DNA analysis, i.e., amplification and profiling, but at the same time can be used to visualize fingerprints.

With Diamond™ Nucleic Acid Dye, we achieve all aspects of the analysis we need—high contrast and low toxicity, which is assumed due to the way the dye binds to DNA (Figure 4 [53,54])—but the binding mechanism and its influence will be extended in further research.

There is no need for harmful reagents and UV light sources for visualization, the material is undamaged for further analysis, and most importantly, the evidential value of the material is increased by performing analysis of DNA extracted from fingerprints with simultaneous visualization of minutiae.

## 4. Conclusions

Further development of techniques for handling touch DNA will contribute to increasing the efficiency of tests leading to the obtaining of a DNA profile, which will be reflected in the benefits and outcomes of criminal proceedings. Most of the articles cited indicated a strong need for continued research and development of new methods that would address the issues discussed for DNA samples.

In our research, we present a method for visualizing fingerprints on non-porous surfaces with the simultaneous possibility of performing DNA analysis from dactyloscopic traces. In addition, there is no need to use multiple fluorescent dyes. The proposed Diamond™ Nucleic Acid Dye provides an alternative to previously used dyes for visualizing dactyloscopic traces on non-porous surfaces with the possibility of performing DNA analysis. The performance of further stages of DNA analysis using our procedure has been verified by spectroscopic measurements of DNA concentration and direct PCR reaction, which suggests that the acquisition/amount of DNA material is sufficient for further investigation, such as profiling. It should be noted that the correct control of fluorescent dyes in the field of molecular spectroscopy has a significant impact on further steps of DNA analysis and, in this case, made it possible to analyze dactyloscopic traces simultaneously. Diamond™ Nucleic Acid Dye is suitable for dual evidence evaluation—friction ridge analysis and DNA from dactyloscopic traces—significantly increasing the evidential value. Furthermore, the spectroscopic and application studies presented, using fingerprints on glass, clearly indicate the major potential of the presented research for the construction of innovative optical sensors, taking into account the current needs of Forensic Science.

## Figures and Tables

**Table 1:** Reagents used in the study.

Product	Purchased From	
Diamond™ Nucleic Acid Dye	Promega Corporation	Madison, WI, USA
Deoxyribonucleic acid from herring sperm	Sigma-Aldrich	Darmstadt, Germany
TBE buffer	Chempur	Silesia, Poland
Direct Tissue PCR Kit	EURx	Gdansk, Poland

**Table 2:** Integrated area value of Diamond™ Nucleic Acid Dye spectra.

Sample	Integrated Area
Reference DNA	12.90
Fingerprint DNA	8.95
Fingerprint DNA after PCR	14.95

## Data Availability

Data of absorption and fluorescence measurement are deposited at Czarnomska, M.; Lewkowicz, A. (2024). Absorption and fluorescence spectrum the Diamond™ nucleic acid dye applied to DNA and friction ridge analysis from fingerprint traces (1–) [Dataset]. Gdańsk University of Technology. https://doi.org/10.34808/dar5-wv41.
